# A canine macrophage cell line that can be polarized provides a model for canine macrophage differentiation

**DOI:** 10.1038/s41598-026-52770-7

**Published:** 2026-05-29

**Authors:** Qingkang Lyu, Victor P. M. G. Rutten, Ildiko Van Rhijn, Willem van Eden, Alice J. A. M. Sijts, Femke Broere

**Affiliations:** 1 Department of Biomolecular Health Sciences, Faculty of Veterinary Medicine, Utrecht University, 3584 CL Utrecht, The Netherlands; 2 Department of Veterinary Tropical Diseases, Faculty of Veterinary Science, Pretoria University, Pretoria, South Africa; 3 Department of Clinical Sciences of Companion Animals, Faculty Veterinary Medicine, Utrecht University, Utrecht, The Netherlands; 4 Immunology Center of Georgia, Augusta University, Augusta, GA USA; 5 Division of Rheumatology, Immunity and Inflammation, Harvard Medical School, Brigham and Women’s Hospital, Boston, MA 02115 USA

**Keywords:** 030D macrophage cell line, Canine macrophage polarization, M1 and M2 macrophage surface marker, Cytokine and chemokine, Phagocytosis, Cell biology, Immunology

## Abstract

Monocyte-like cell lines are valuable models for studying macrophage biology, yet a well-characterized canine macrophage model has been lacking. The canine histiocytic sarcoma-derived cell line 030D was assessed for its macrophage polarization potential by stimulation with IFN-γ + LPS (M1) or IL-4 (M2), or no additives (M0) and characterized for morphology, immunophenotype, marker gene expression, and phagocytic capacity. 030D M1 macrophages expressed higher levels of the surface markers CD32, CD40, CD80, CD83, CD86 and MHC II than 030D M2 macrophages. qPCR analysis showed higher expression of pro-inflammatory cytokines (IL-6, IL-1β, TNF-α, IL-12p35/40, IL-23p19, COX2, CCL2, and CCR7), anti-inflammatory cytokines TGF-β and IL-10, as well as higher expression of LOX-1, LXN, and arginase-1 by 030D M1 macrophages compared to 030D M2 macrophages. Conversely, established M2 markers MS4A2 and CD206 were significantly higher in 030D M2 macrophages compared to 030D M1 macrophages. Functionally, 030D M1 macrophages displayed higher phagocytic activity than 030D M2 macrophages. Comparison of 030D-derived macrophages with primary canine monocyte-derived macrophages (MDMs) demonstrate that both models, with the exception of TGF-β and IL-10, resemble each other in the studied parameters. Thus, the 030D cell line provides a highly accessible and validated tool for studying canine macrophage biology and immune responses.

**Supplementary Information:**

The online version contains supplementary material available at 10.1038/s41598-026-52770-7.

## Introduction

Macrophages are pivotal components of the innate immune system, known for their plasticity and diverse functional roles. As professional phagocytes, they eliminate pathogens and cellular debris while regulating immune responses, maintaining tissue homeostasis, and facilitating tissue repair. Their functional diversity is driven by polarization into distinct phenotypes, including classically activated macrophages (M1) and alternatively activated macrophages (M2). In human disease and mouse models thereof, M1 macrophages are pro-inflammatory and critical for control of infections and mediating anti-tumor immunity, whereas M2 macrophages are involved in wound healing, tissue remodeling, and immune regulation^1^. This ability to polarize reflects macrophages’ capacity to adapt to microenvironmental cues, making them central players in health and disease.

In the canine immune system, macrophages perform similarly critical roles in disease processes, including chronic inflammation, autoimmunity, infection, and cancer. M1 macrophages play a critical role in exacerbating common canine inflammatory conditions such as arthritis, chronic dermatitis, and systemic lupus erythematosus^2,3^. These macrophages are characterized by their secretion of pro-inflammatory cytokines, including tumor necrosis factor-alpha (TNF-α), interleukin-6 (IL-6), and interleukin-1β (IL-1β), and their expression of surface markers such as CD80, CD86, and major histocompatibility complex class II (MHC II)^4,5^. Conversely, M2 macrophages, or alternatively activated macrophages, are associated with immune regulation, tissue remodeling, wound healing, but also with tissue fibrosis^6,7^. They are known to produce anti-inflammatory cytokines such as interleukin-10 (IL-10) and transforming growth factor-beta (TGF-β), and they express surface markers like CD206 (mannose receptor) and arginase-1^4,5^. Conversely, in canine cancers and infections, M2 are typically permissive to growth and immune evasion of parasites, bacteria, or cancer cells whereas M1 contribute to clearance.

Research on canine macrophage polarization or research using polarized macrophages is hampered by scarcity of suitable in vitro models. Macrophages are cultured from various murine tissues, including bone marrow, peripheral blood mononuclear cells, peritoneum and spleen^8^. However, obtaining these materials from dogs presents significant ethical challenges. In other species, monocyte-like cell lines have been widely applied as a powerful model for the investigation of immune responses in vitro^9^, with distinct advantages such as ease of culture, scalability, and homogeneity^10^. Well-established macrophage cell lines such as THP-1, HL-60, MonoMac-6, and U937 cell (human), RAW264.7, BAC1.2F5, and J774.1 cell (mouse), and HD11 cell (chicken), have proven essential for functional and translational immunological studies^9,11–13^. Therefore, use of canine mononuclear phagocytic cell lines would offer significant opportunities to advance our understanding of canine macrophage biology. The canine histiocytic sarcoma cell line DH82 has the ability to differentiate and polarize into M1 and M2 macrophages, although the phenotypic differences between the two states has not been fully explored^14–16^. Having access to additional, characterized canine cell line-based models for macrophage biology would advance both basic and translational studies in canine health.

In this study, we used the canine monocyte-like cell line 030D, which was established by Gebhard et al. (1995) from a dog diagnosed with malignant histiocytosis^17^. 030D cells have been instrumental in studies of canine immunology and infection^18–21^. However, their potential for differentiation and polarization into functional M1 and M2 macrophages has yet to be comprehensively characterized. To assess this, 030D cells (M0) were stimulated with IFN-γ + LPS for M1 polarization or IL-4 for M2 polarization. We examined morphological differences during polarization, assessed the expression of M1 and M2 surface markers and cytokine genes, and evaluated phagocytic activity using latex beads. Our findings demonstrate that 030D-derived macrophages can be reliably polarized into M1 and M2 macrophages, establishing these cells as a valuable tool for studying canine macrophage biology, for research into macrophage-driven diseases in dogs and for the development of targeted immunotherapies and treatments in dogs.

## Results

### Morphological changes of 030D-derived macrophages during polarization

**Fig. 1:**
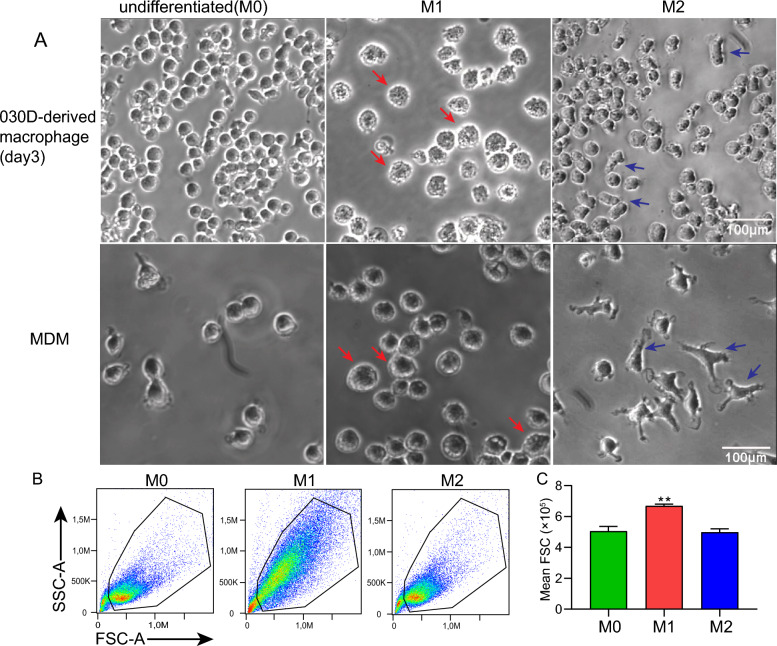
Morphological changes in polarized 030D macrophages and canine MDMs. For the M0 state, undifferentiated canine 030D cells or primary monocytes were cultured with complete medium only. Polarization conditions are described in Materials and Methods. (**A**) representative phase-contrast microscopy images show morphological changes of 030D-derived macrophages at day 3 (top row) and monocyte-derived macrophages (MDMs) at day 7 (bottom row). Large, rounded, or amoeboid morphology is indicated with red arrows, whereas elongated irregular shapes are indicated with blue arrows. Scale bar = 100 μm. (**B**) Representative flow cytometry plots of forward scatter (FSC) versus side scatter (SSC) obtained from 030D-derived M0, M1, and M2 macrophages at day 3. (**C**) Mean FSC values were quantified across polarization states. Data are presented as mean ± SD, with n ≥ 3 per condition. *p < 0.05; **p < 0.01.

To determine whether the canine macrophage cell line 030D can be polarized into the classically activated (M1) and the alternatively activated (M2) macrophage phenotypes, 030D cells were stimulated with IFN-γ + LPS (M1) or IL-4 (M2) for 3 days. As an initial means to investigate macrophage polarization, morphological changes of the cells were monitored daily to evaluate polarization. Undifferentiated 030D cells maintained a consistent, small and round morphology throughout the 3-day culture period, as shown in Figs. 1 and S1. In contrast, M1-polarized macrophages exhibited significant morphological changes, including a well-spread, large and flat shape, with some cells adopting an amoeboid “fried-egg” morphology (Fig. 1A, red arrows). Flow cytometry analysis revealed increased forward scatter (FSC) values, indicating that M1 cells were larger and more granular than undifferentiated macrophages from day 2 onwards (Figs. 1B, C, S1). M2-polarized 030D macrophages remained small and round on days 1 and 2 (Fig. S1) but developed an elongated, spindle-like morphology by day 3 (Fig. 1A, blue arrows). Unlike M1 macrophages, M2 cells did not exhibit a significant increase in size relative to undifferentiated 030D cells.

To study the potential usefulness of polarized 030D-derived macrophages as a model system, we compared them with polarized canine primary MDMs. M1 MDMs differentiated into bigger, round cells, predominantly displaying an amoeboid “fried-egg” shape, while M2 macrophages acquired an elongated spindle-like morphology (Fig. 1A). These observations mirror our findings in 030D-derived macrophages, highlighting their morphological similarity to primary canine M1 and M2 MDMs.

### Cell surface marker expression of canine 030D-derived macrophages

**Fig. 2:**
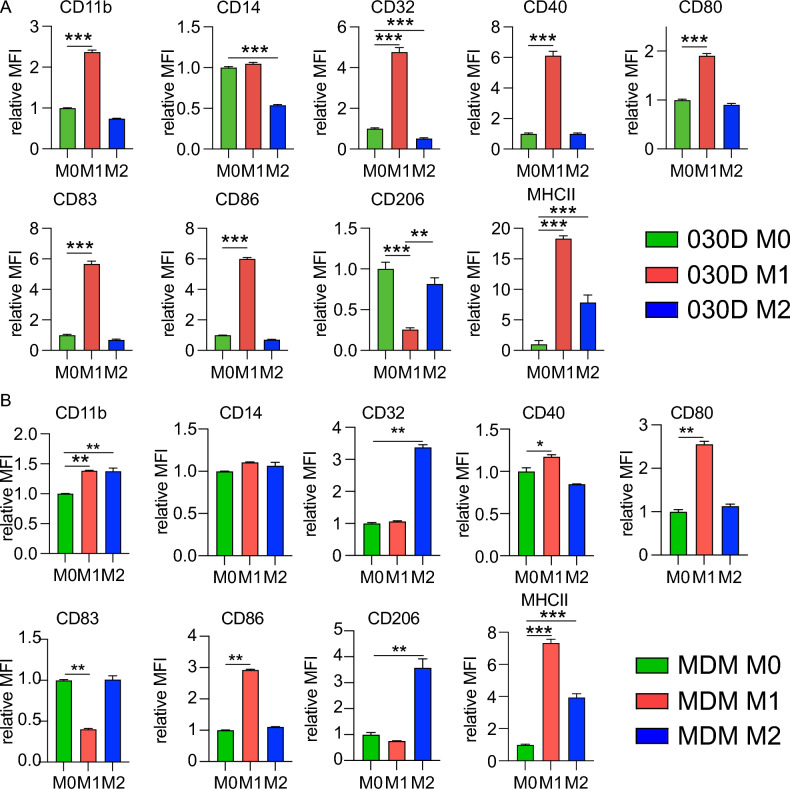
Flow cytometric analysis of surface markers expressed in 030D-derived macrophages and MDMs. (**A**) 030D cells were polarized as described in materials and methods for 3 days and (**B**) canine primary MDMs for 7 days. Expression of surface markers by 030D-derived macrophages was evaluated by flow cytometry. Relative mean fluorescence intensity (MFI) was calculated by first subtracting the corresponding FMO control from the geometric MFI for each sample, and subsequently normalizing the values for M1 and M2 cell to M0 cells. Data are presented as mean ± SD, with n = 3 per condition. *p < 0.05; **p < 0.01, ***p < 0.001. MDM data represent three independent dog donors (not pooled). 030D data represent three independent experiments.

To characterize the polarized states, we analyzed surface marker expression in 030D-derived macrophages. Pan-macrophage markers CD14 and CD11b were expressed across undifferentiated, M1, and M2 macrophages (Fig. 2A, S2). M1-polarized 030D macrophages exhibited elevated levels of CD32, CD40, CD80, CD83, CD86, and MHC II, with significant upregulation from day 2 onwards (Figs. 2A, S2). Despite low baseline expression, MHC II was notably higher in M1 macrophages but also increased in M2 cells. Conversely, CD206, which was previously reported as an M2 marker^27–29^, decreased in both M1 and M2 macrophages compared to M0, with a more pronounced reduction in M1 cells.

Polarized canine primary MDMs (Fig. 2B) show a pattern where M1 MDMs expressed higher levels of CD40, CD80, CD86, and MHC II compared to M0 MDMs. M2 MDMs showed increased expression of CD32 and CD206, while CD83 was reduced in M1 but unchanged in M2 cells. These findings align with our previous study on canine MDMs^30^.

Collectively, these results demonstrate that 030D-derived macrophages can be polarized to M1 and M2 phenotypes. A comparison between polarized 030D macrophages and polarized canine primary MDMs shows some notable differences: CD83 is high in 030D-derived M1 macrophages compared to M0 and M2, and low in M1 MDMs compared to M0 and M2. In other species CD83 is higher on M1 than on M2, so for this marker the 030D-derived macrophages are more constant with other species’ macrophages than our MDM. Another difference is that CD32 is high on 030D-derived M1 macrophages compared to M0 and M2, but among the MDMs it is the M2 population that expresses most CD32. However, the monoclonal antibody we used does not distinguish between CD32a and CD32b, which are differentially expressed in M1 (CD32A > CD32B) versus M2 macrophages (CD32B > CD32A)^31,32^, so it is undetermined if our CD32 results really reflects an opposite expression pattern. The other studied cell surface markers (CD40, CD80, CD86, CD206, and MHC II) show similar patterns of cell surface markers by the different polarization states when comparing 030D macrophages to polarized canine primary MDMs.

### Cytokine and chemokine gene expression in 030D-derived macrophages

**Fig. 3:**
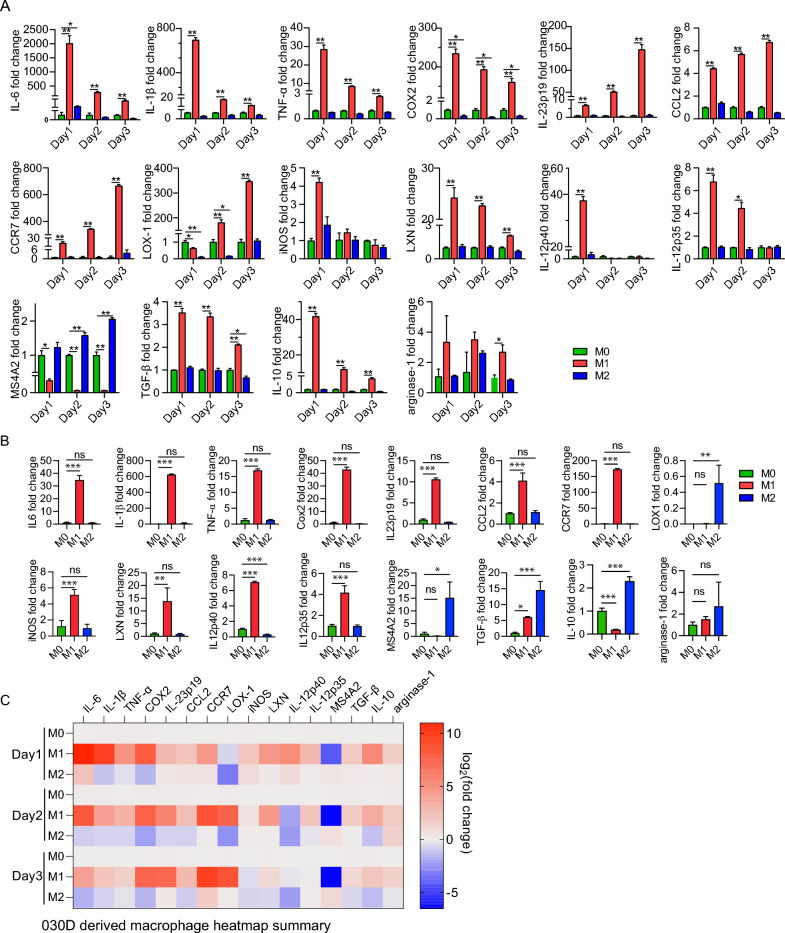
Cytokine and chemokine expression profiles in 030D-derived macrophages and MDMs. Canine 030D-derived M0, M1 and M2 macrophages were collected at day 1, 2 and 3, and canine monocyte-derived macrophage were collected at day 7. Total mRNA was extracted, reverse-transcribed into cDNA, and analyzed for cytokine and chemokine expression by qPCR. (**A**) Cytokine and chemokine expression in 030D-derived macrophages at days 1–3. (**B**) Cytokine and chemokine expression in canine monocyte-derived macrophages at day 7. M0 macrophages were used as the control group. Gene expression levels were normalized to housekeeping genes RPS19. (**C**) Heatmap summary of cytokine and chemokine expression in 030D-derived macrophages at days 1–3, with the color scale representing log₂ (fold change). iNOS: inducible nitric oxide synthase; MS4A2: Membrane Spanning 4-Domains A2; LOX-1: the lectin-like oxidized low-density lipoprotein receptor-1; LXN: Latexin. Data are presented as mean ± SD, with n = 3 per condition. *p < 0.05; **p < 0.01, ***p < 0.001. MDM data represent three independent dog donors (not pooled). 030D data represent three independent experiments.

To further characterize canine 030D-derived macrophages, we measured the mRNA levels of cytokines and chemokines known to be associated with M1 and M2 polarization states. M1-polarized macrophages exhibited significantly elevated expression of classical M1 marker genes, including IL-6, IL-1β, TNF-α, COX2, iNOS, IL-12p35 and IL-12p40, at day 1 (*p* < 0.01). These levels declined progressively at day 2 and 3 (Fig. 3A). In contrast, these genes were not upregulated in M2-polarized macrophages during the same time frame. Additional M1-associated genes, such as IL-23p19, CCL2, and CCR7, were also significantly upregulated in M1-polarized macrophages at day 1 (*p* < 0.01) and their expression levels continued to increase in subsequent days (Fig. 3A). The expression of two emerging M1 markers, lectin-like oxidized low-density lipoprotein receptor-1 (LOX-1) and latexin (LXN), was markedly higher in M1-polarized macrophages compared to undifferentiated cell and M2 macrophages, consistent with findings in previous studies on both canine and human macrophages^22,27^. In contrast, a newly proposed M2 marker^27^, the beta subunit of the high affinity IgE receptor membrane spanning 4-domains A2 (MS4A2) expression was significantly upregulated in M2-polarized macrophages, while its expression was reduced in M1-polarized macrophages compared to undifferentiated cells (Fig. 3A). Interestingly, other genes that are typically associated with M2 macrophages, such as TGF-β, IL-10, and arginase-1, exhibited higher expression in M1-polarized macrophages than in M2-polarized cells.

Similarly, in canine M1 MDMs, M1-associated genes such as IL-6, IL-1β, TNF-α, COX2, IL-23p19, CCL2, CCR7, iNOS, LXN, IL-12p35 and IL-12p40 were significantly upregulated, paralleling the expression patterns observed in 030D-derived M1 macrophages. In canine M2 MDMs, the M2-associated gene MS4A2 showed increased expression, consistent with 030D-derived M2 macrophages. Notably, LOX-1 expression was higher in 030D-derived M1 cells than M2 cells, while it was elevated in M2 MDMs compared to M1 MDMs. Furthermore, no significant changes in arginase-1 expression were observed between undifferentiated monocytes, M1 MDMs, and M2 MDMs.

While both models demonstrate clear M1 and M2 phenotypic signatures, and our comparative mRNA analysis highlights that 030D-derived M1 and M2 macrophages exhibit similar cytokine and chemokine profiles to M1 and M2 MDMs, TGF-β and IL-10 are atypically expressed by the M1 macrophages in the 030D model.

### Enhanced phagocytic capacity in canine 030D-derived macrophages

**Fig. 4:**
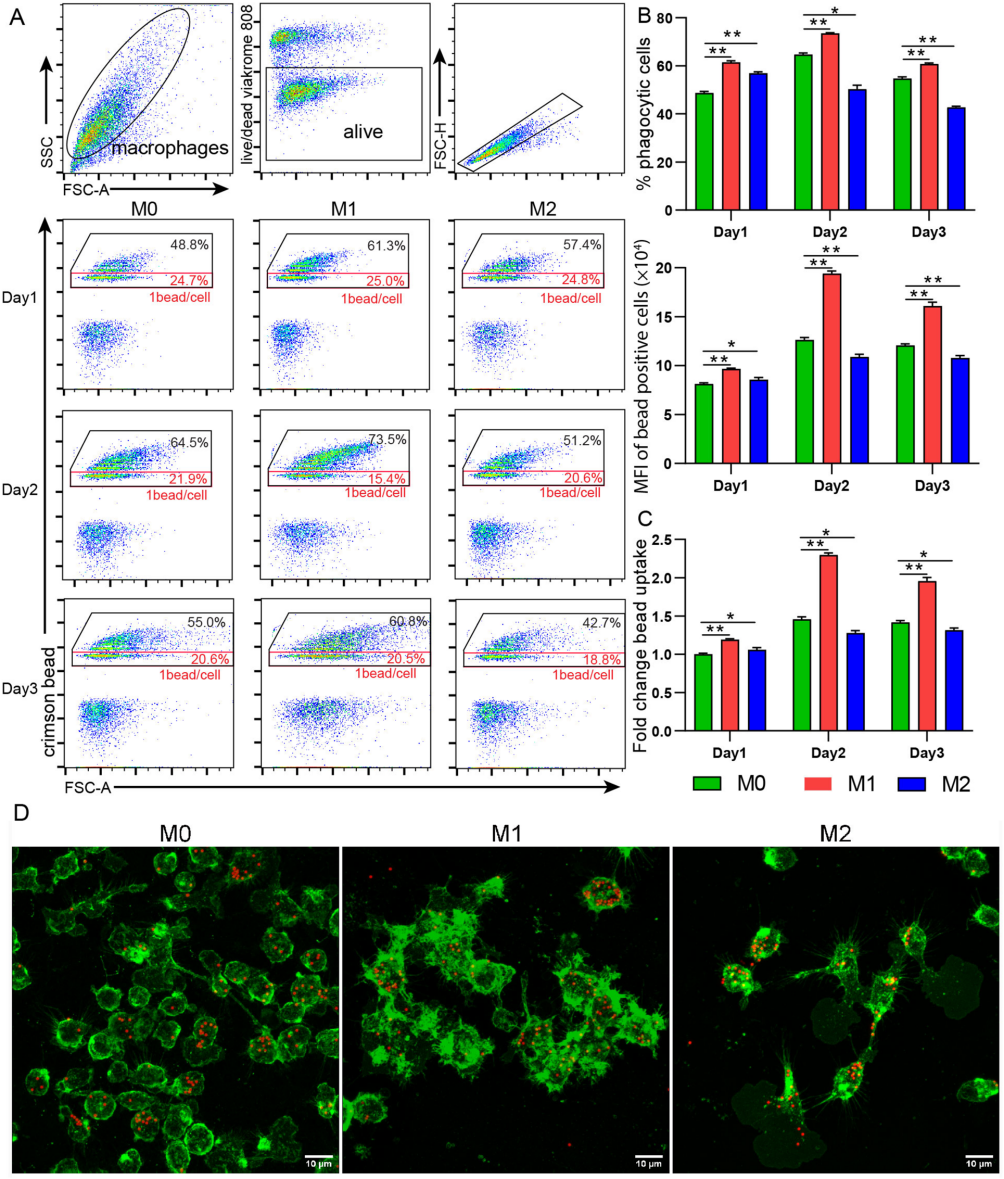
Phagocytic activity of canine 030D-derived macrophages. 030D macrophages were polarized for 1, 2 or 3 days , followed by incubation with crimson carboxylate-modified FluoSpheres fluorescent beads. Phagocytic activity was quantified using flow cytometry. (**A**) Gating strategy: macrophages were selected based on scatter profile (FSC/SSC), viability staining (ViaKrome), and singlet discrimination. Representative dot plots show bead uptake at each time point. Cells containing a single bead were gated in red, whereas cells with multiple beads were gated in black, from which the average bead uptake was calculated. (**B**) Phagocytic activity is presented as the percentage of bead-positive cells (top) and median fluorescence intensity (MFI) (bottom). (**C**) Fold change in bead uptake by M1- and M2-polarized macrophages was calculated relative to M0 macrophages. (**D**) Representative confocal images of day 2 macrophages stained with wheat germ agglutinin (WGA, green) to visualize the plasma membrane. Internalized FluoSpheres fluorescent beads appear in red. Images show 030D-derived M0, M1 and M2 macrophages; corresponding 3D reconstructions based on z-stack are provided in Supplementary Videos S2A-S2C. Data are presented as mean ± SD, with n ≥ 3 per condition. *p < 0.05; **p < 0.01. Scale bar = 10 μm. 030D data represent three independent experiments.

Phagocytosis is a key functional characteristic of macrophages. To evaluate this capacity in canine 030D-derived macrophages, we assessed the uptake of fluorescent latex beads by undifferentiated 030D cells, and M1- and M2-polarized macrophages over three days. All polarization states displayed robust bead uptake at days 1, 2, and 3 (Fig. 4). Notably, M1-polarized macrophages exhibited the highest phagocytic activity, as indicated by both the percentage of bead-positive cells and the mean fluorescence intensity (MFI), which were significantly higher (*p* < 0.01) compared to undifferentiated cells and M2 macrophages at all time points (Fig. 4A, B). Phagocytic activity peaked at day 2 for both M0 cells and M1 macrophages. In contrast, M2-polarized macrophages showed a gradual decrease in the proportion of bead-positive cells from day 1 to day 3. However, the MFI and fold change in bead uptake increased within the M2 populations, indicating a higher number of beads per phagocytic M2 macrophage (Fig. 4B-C). As expected, control macrophages incubated with beads at 4 °C failed to engulf beads, confirming the specificity of phagocytosis (data not shown).

Confocal imaging with plasma membrane staining demonstrated that a substantial fraction of the beads was localized in the cytoplasm of M0, M1, and M2 macrophages at all time points, while only very few beads were attached to the surface of the macrophages (Figs. 4D, S3, and Videos 1–3). These findings emphasize the superior phagocytic ability of M1-polarized macrophages and reveal dynamic changes in bead uptake across macrophage subsets during polarization.

## Discussion

Macrophage polarization is essential for immune regulation, influencing inflammation, tissue repair, and pathogen defense. Although monocyte-like cell lines are widely used to study murine and human macrophage biology, only one canine macrophage cell line, DH82, has been partly characterized^15,16^. In the current study we evaluated the canine 030D cell line as an in vitro model for macrophage polarization. We established a differentiation protocol and characterized undifferentiated 030D cells (M0) and their polarized M1 and M2 counterparts in terms of morphology, surface marker expression, cytokine profiles, and phagocytic properties. Our findings demonstrate that 030D-derived macrophages can be differentiated to exhibit distinct M1- or M2-like phenotypes, gene expression patterns, and functional differences that recapitulate aspects of canine MDMs. Nevertheless, the 030D cell line is derived from canine histiocytic sarcoma and therefore represents a transformed cell model. Although 030D-derived macrophages reproduce key features of macrophage polarization, some differences were observed, as discussed below, and additional differences should be expected in parameters that were not measured in the current study.

In this study, 030D-derived M1 macrophages exhibited a large, round, and amoeboid shape, whereas a subset of M2 macrophages developed an elongated, spindle-like morphology. These findings align with previous studies reporting distinct morphological features characteristic of M1 and M2 phenotypes in other species^22–26^. In contrast, the canine DH82 macrophage cell line was previously reported to show less clear morphological distinction between M1 and M2 polarization states, as both DH82-derived subsets are rather diverse and include round, amoeboid, and large/flat morphology^15,16^. A spindle-like morphology was only observed in late-passage, undifferentiated DH82 cells (passage ≥ 66), likely due to genetic drift and functional alterations from prolonged subculturing^16^. Unlike DH82 cells, the 030D cell line consistently exhibited clear morphological differences between M1 and M2 polarization states, highlighting their suitability as a polarization model.

Consistent with macrophage polarization patterns observed in human and murine models^33,34^, 030D-derived M1 macrophages exhibited upregulation of CD32, CD40, CD80, CD83, CD86, and MHC II at the cell surface, whereas M2 macrophages displayed elevated expression of CD206. Of note, MHC II levels at the cell surface of 030D cells were quite low at baseline so the significant upregulation under polarizing conditions led only to modest levels of MHC II. A comparison with the published literature on the DH82 canine macrophage cell line could only be made for CD80 and CD206: like 030D-derived M1 macrophages, DH82-derived M1 macrophages showed increased expression of CD80^15,16^. CD206 expression in DH82-derived M2 macrophages was not significantly different from M1 macrophages^15^.

Macrophage polarization is also associated with distinct cytokine and chemokine expression patterns. M1 macrophages are characterized by a strong pro-inflammatory response, with elevated levels of mRNA encoding IL-6, IL-1β, TNF-α, COX2, iNOS, IL-12p35, IL-12p40, IL-23p19, CCL2, and CCR7, mirroring cytokine profiles observed in M1 MDMs. The more recently identified M1 markers, LOX-1 and LXN were also highly upregulated, further supporting their classification as M1-associated genes^22,27^. Interestingly, while LOX-1 was predominantly expressed in 030D-derived M1 macrophages, it was more highly expressed M2 MDMs, suggesting potential cell-line-specific regulatory differences.

030D-derived M2 macrophages, expected to exhibit an anti-inflammatory profile, showed increased MS4A2 expression, consistent with M2 MDMs^27,30^. However, TGF-β and IL-10, were higher in 030D-derived M1 macrophages than in M2^35,36^. This phenomenon has been reported in human macrophages, where IL-10 production is not strictly confined to M2 cells, highlighting the complexity of cytokine regulation during polarization^37,38^. The higher TGF-β and IL-10 in 030D-derived M1 macrophages compared to in M2 is somewhat inconsistent with results obtained in primary canine MDMs, which showed increased expression of these regulatory cytokines under M2-polarizing conditions. This apparent discrepancy likely reflects fundamental differences between transformed cell lines and primary macrophages. The 030D cell line originates from malignant histiocytic cells and may therefore exhibit altered baseline signaling and cytokine regulation compared to primary cells, as reported for other macrophage-like cell lines^26,39^. IL-10 can also be produced by pro-inflammatory macrophages as part of a negative feedback mechanism to limit excessive inflammation^40^. Similarly, TGF-β expression is influenced by activation state, cellular origin, and environmental cues. Although 030D-derived M1 macrophages exhibited a slightly higher expression of iNOS, nitric oxide (NO) production was undetectable in any of the polarization states by the Griess assay (data not shown). This is consistent with previous studies on DH82 cells, which also failed to produce detectable levels of NO upon activation^41–43^, although it is possible that 030D-derived macrophages generate NO at levels below the detection limit of the Griess assay.

Phagocytosis is a key macrophage function influenced by polarization state. In contrast to MDMs, where M2 macrophages exhibited superior phagocytic activity, 030D-derived M1 macrophages demonstrated the highest bead uptake, as evidenced by both the percentage of bead-positive cells and mean fluorescence intensity^30^. This discrepancy may arise from differences in receptor expression and the preferred mechanism by which latex beads are phagocytosed by macrophages: M1 macrophages in the 030D model exhibited higher levels of CD32, which mediates FcγR-dependent phagocytosis, while M2 macrophages expressed CD206, which facilitates glycolipid- and glycoprotein-mediated phagocytosis^44–49^.

Our findings further support the notion that macrophage polarization is not a rigid dichotomy but rather a continuum with partly overlapping features. A study in human skeletal muscle macrophages identified cells expressing both M1 and M2 markers, including CD11b, CD14, CD68, CD86, and CD206, demonstrating that macrophages can simultaneously exhibit characteristics of both polarization states^50^. Macrophage plasticity allows for dynamic and reversible phenotypic transitions in response to environmental cues. For in vitro scientific experiments, understanding the different models and their flexibility enables scientist to choose a macrophage model that represents critical aspects of their research question.

In conclusion, this study developed a polarization protocol for the canine macrophage cell line 030D, generating M1 and M2 macrophages with distinct morphology, phenotypic profiles, gene expression, and function. Despite some differences, the similarities between 030D-derived macrophage and polarized primary canine monocytes establish the utility of the 030D cell line as a reliable and scalable model for studying canine macrophage biology.

## Materials and methods

### 030D Cell culture and polarization

The 030D canine monocytoid cell line was originally derived from canine malignant histiocytosis and generously provided by D. Gebhard^17^. The cells were maintained in RPMI-1640 GlutaMAX (Life TechnologiesTM Ltd., Paisley, Scotland, UK) supplemented with 5% fetal bovine serum (BODINCO B.V., The Netherlands), and 1% penicillin–streptomycin (Life TechnologiesTM Ltd., Paisley, Scotland, UK). Cultures were incubated at 37 °C in a humidified atmosphere with 5% CO_2_. 030D cells were used within three passages after thawing from the same batch, to minimize variability associated with prolonged culture.

To induce polarization, 030D cells were seeded in 6-well plates at a density of 8 × 10^5^ cells/well. After a 2 h incubation, the medium was gently replaced to remove non-adherent cells. For M1 polarization, 030D cells were stimulated with 20 ng/mL recombinant canine IFN-γ (R&D system, Abingdon, United Kingdom) for 6 h, followed by the addition of 100 ng/L lipopolysaccharide (LPS) from Escherichia coli O111:B4 (Sigma-Aldrich, Saint Louis, MO, USA). To generate M2 macrophages, 030D cells were cultured with 40 ng/mL recombinant canine IL-4 (R&D system, Abingdon, United Kingdom). Cells maintained in complete medium without additional stimuli were designated as M0 macrophages. The morphology of 030D M0, M1, and M2 macrophages was observed at 24, 48, and 72 h using phase-contrast light microscopy with a Nikon Eclipse TS100 inverted microscope (Nikon Instruments Europe B.V., Amstelveen, The Netherlands) and ImageFocus software (v3.0.0.2). Images were processed using Fiji ImageJ. After the specified culture periods, macrophages from all groups were harvested for downstream analyses.

### Isolation and polarization of primary canine monocytes

Blood samples and buffy coats were obtained from excess blood drawn for the canine blood bank and diagnostic purposes, which was not considered experimentation on animals by the Animal Ethics Committee from Utrecht University. The blood samples were from male and female dogs (aged 2–8 years, various breeds) with informed owner consent, through the Companion Animal Clinics at the Faculty of Veterinary Medicine, Utrecht University. Sample collection was performed by licensed veterinarians from the Department of Clinical Sciences of Companion Animals. Each donor contributed no more than once every six months. For primary canine monocyte-derived macrophages (MDMs), a total of 6 individual dog donors were included across the study. MDMs were derived from independent donors and were not pooled. Specifically, three donors were used for experiments shown in Fig. 2, and three donors for Fig. 3.

Peripheral blood mononuclear cells (PBMCs) were isolated from buffy coats from approximately 30 mL of blood, by density gradient centrifugation using Histopaque-1077 (Sigma Aldrich, UK). Buffy coats were diluted 1:1 with phosphate-buffered saline (PBS) and carefully layered over an equal volume of Histopaque-1077. The samples were centrifuged at 800 × g for 30 min at room temperature without braking. The PBMC layer at the interface was collected and washed three times with PBS supplemented with 0.5% fetal calf serum (FCS) and 2 mM EDTA (MACS buffer). Monocytes were then isolated using magnetic-activated cell sorting (MACS) with a mouse anti-CD14 antibody followed by anti-mouse IgG magnetic microbeads (Miltenyi Biotec, Germany), according to the manufacturer’s standard protocol.

Isolated CD14⁺ monocytes were seeded into 24-well plates at a density of 7.5 × 105 cells per well in 1 ml of RPMI-1640 GlutaMAX medium (Life Technologies, UK). The medium was supplemented with 5% fetal bovine serum (BODINCO, The Netherlands) and 1% penicillin/streptomycin (Life Technologies, UK). Cells were cultured at 37 °C in a humidified incubator with 5% CO₂.

To generate monocyte-derived macrophages (MDMs), CD14⁺ monocytes were treated with either recombinant canine GM-CSF (R&D Systems, UK) or recombinant human M-CSF (Gibco, Thermo Fisher Scientific, USA), with media refreshed every other day. On day 5, cells in the GM-CSF group were polarized to an M1 phenotype by adding 20 ng/mL recombinant canine IFN-γ (R&D Systems, UK) and 100 ng/mL lipopolysaccharide (LPS) from Escherichia coli O111:B4 (Sigma-Aldrich, USA). Cells in the M-CSF group were polarized to an M2 phenotype by adding 40 ng/mL recombinant canine IL-4 (R&D Systems, UK).

A control group (M0) was maintained under identical culture conditions without the addition of cytokines or stimulants, representing monocytes in culture, as opposed to freshly isolated monocytes. All MDMs—M0, M1, and M2—were harvested on day 7 for downstream analyses, including phenotypic characterization, mRNA extraction, and phagocytosis assays.

### Phenotypic characterization of 030D-derived and MDMs by flow cytometry

For flow cytometry analysis, 030D M0, M1 and M2 macrophages were collected at days 1, 2, and 3 and MDMs at day 7. Cells were washed with cold PBS and detached by gentle scraping before being transferred into a 96-well round-bottom plate at a density of 2 × 10^5^ cells/well. To minimize non-specific antibody binding, Fc receptors were blocked using 10% autologous dog serum for 30 min. Macrophages were stained on ice for 30 min with antibodies targeting CD14, CD40, CD80, MHC II, CD206, CD83, CD86, CD32, and CD11b (see Table 1 for details). Following staining, cells were washed 3 times with FACS buffer (2% FCS in PBS) and, when required, incubated with secondary or streptavidin-conjugated antibodies for an additional 30 min on ice. Dead cells were excluded using ViaKrome 808 Fixable Viability Dye (Beckman Coulter, Woerden, Netherlands).

**Table 1:** List of antibodies used for flow cytometry.

Antigen, conjugate	Original target species	Reference for canine reactivity	Clone	Isotype	Dilution	Source
CD14, vioblue	Human	^51^	TÜK4	mouse IgG2a κ	1:100	Miltenyi Biotec
CD11b, biotin	Dog	^52^	CA16.3E10	mouse IgG1	1:100	P. Moore*
CD32A + B biotin	Human	^53^	AT10	mouse IgG1	1:50	Abcam
CD40, R-PE	Human	^54^	LOB7/6	mouse IgG2a	1:25	BIO-RAD
CD80, FITC	Mouse	^55^	16-10A1	hamster IgG2	1:100	BD Biosciences
CD86, unconjugated	Dog	^55^	CA24.3E4	mouse IgG1	1:50	P. Moore*
CD83, APC	Human	^30^	HB15e	mouse IgG1, κ	1:50	Biolegend
CD206, APC-Cy7	Human	^15^	15–2	mouse IgG1, κ	1:50	Biolegend
MHC II, APC	Dog	^56^	YKIX334.2	rat IgG2a, κ	1:50	eBioscience™
GaM-IgG1-PerCP	Mouse		–	polyclonal IgG	1:100	Santa Cruz Biotech
streptavidin -PE	–		–	–	1:2000	BD Biosciences

*Peter Moore, University of California, Davis, CA, USA.

A minimum of 15,000 events per sample were acquired using a CytoFLEX LX flow cytometer (Beckman Coulter, CA, USA). The acquired data were analyzed using FlowJo software version 10.5 (FlowJo LLC, Ashland, USA) to evaluate the phenotypic profiles of the macrophages. Relative mean fluorescence intensity (MFI) was calculated by first subtracting the corresponding FMO control from the geometric MFI for each sample, and subsequently normalizing the values for M1 and M2 cells to M0 cells.

### Gene expression analysis of 030D-derived- and MDMs by real-time qPCR

To evaluate gene expression, 030D-derived macrophages were harvested on days 1, 2 and 3 and MDMs on day 7, in 350 μl of RLT lysis buffer (Qiagen, Venlo, The Netherlands). Total RNA was extracted using the Qiagen RNeasy mini kit (Qiagen, Venlo, The Netherlands) according to the manufacturer’s instructions and then treated with DNase I to remove genomic DNA contamination. RNA was dissolved in RNase-free water, and concentrations were measured using a NanoDrop-1000 Spectrophotometer (Isogen Lifescience B.V., Utrecht, the Netherlands). cDNA was synthesized from 1 μg of total RNA using the iScript cDNA Synthesis Kit (Bio-Rad Laboratories B.V., California, USA) following the manufacturer’s instructions.

RT-qPCR was performed using the CFX connect real-time system (Bio-Rad Laboratories B.V., Veenendaal, The Netherlands) with iQ SYBR Green Supermix (Bio-Rad Laboratories B.V., California, USA). Data acquisition and analysis were conducted using CFX Maestro 1.1 software. Primers for target genes were obtained from Invitrogen (Life Technologies Ltd, paisley, UK). The sequences of primers are listed in Table 2. GAPDH and RPS19 genes were used as reference genes for normalization of target gene expression. Amplification efficiencies were determined using standard curves generated by serial dilution and calculated using Bio-Rad CFX Maestro software. All primer pairs showed efficiencies within 90–110%. Relative mRNA expression levels were calculated using the Pfaffl-method.

**Table 2:** Sequences of primers used for quantitative real-time PCR.

Gene	Forward primer (5’-3’)	Reverse primer (5’-3’)	Accession number, reference	Amplification efficiency	Product length (bp)
RPS19	CCTTCCTCAAAAAGTCTGGG	GTTCTCATCGTAGGGAGCAAG	DR103251.1	91.8	95
TGF-β	CAAGGATCTGGGCTGGAAGTGGA	CCAGGACCTTGCTGTACTGCGTGT	^57^	91.6	^57^
TNF-α	CCCCGGGCTCCAGAAGGTG	GCAGCAGGCAGAAGAGTGTGGTG	^58^	107.3	^58^
IL-10	CCCGGGCTGAGAACCACGAC	AAATGCGCTCTTCACCTGCTCCAC	^58^	92.6	^58^
IL-1β	TCTCCCACCAGCTCTGTAACAA	GCAGGGCTTCTTCAGCTTCTC	Z70047	97.6	80
IL-6	TCCTGGTGATGGCTACTGCTT	GACTATTTGAAGTGGCATCATCCTT	U12234	106.8	78
GAPDH	CTGAACGGGAAGCTCACTGG	CGATGCCTGCTTCACTACCT	AB038240.1	100.3	129
Arginase-1	GCATGAGTTCCACGGCAAAA	CTTTTTCCACCCCTCCTCGT	XM_532053.6	90.9	81
iNOS	AATGGAGAGTTGGGCCTTCC	TGGCCCTTAAGAGAAGACTGG	NM_001313848.1	92.7	227
IL-12p40	CAGCAGAGAGGGTCAGAGTGG	ACGACCTCGATGGGTAGGC	AF244915	97.9	109
IL-23p19	CAAGGGGAGAAAAACAGCAG	TGCTGTCCGTTCTGTGAGTC	XM_538231	104.4	78
CCL2	CCTGCTGCTATACACTCA	GCTTCTTTGGGACACTTG	U29653	97.9	91
CCR7	TGGTGGTGGCTCTCCTTGTC	AAGTTCCGCACGTCTTTCTTG	XM 548,131.2	100.3	136
IL-12p35	GTGCCTCAACCACTCCCAA	CAATCTCTTCGGAAGTGCAGG	NM_001003293	108.3	100
COX2	TTCCAGACGAGCAGGCTAAT	GCAGCTCTGGGTCAAACTTC	^58^	93.2	112
LXN	CAGGGTCAAGCAAGTGCAAAG	CTTCCTTTGGCAGACGGGTA	XM_038432753.1	93.6	164
MS4A2	CTCCCCACCACCCACTTATG	AACTCAGCTCTCAACCAGCC	XM_038429453.1	93.3	173
LOX-1	CCCAGGAGTCAGAAAGGGAAC	TCCTGGGGACAATGACCTGAA	XM_038439023.1	100.6	157

MS4A2: Membrane Spanning 4-Domains A2; LOX-1: the lectin-like oxidized low-density lipoprotein receptor-1; LXN: Latexin.

### Phagocytosis assay by flow cytometry

The phagocytic capacity of macrophages was evaluated using 1 μm crimson carboxylate-modified FluoSpheres fluorescent beads (Life Technologies Corporation, Eugene, USA). Specifically, macrophages polarized for 24, 48, and 72 h were incubated with the FluoSpheres fluorescent beads at a bead-to-cell ratio of 10:1 for 4 h. After incubation, cells were washed three times to remove non-phagocytosed beads. Harvested cells were washed 2 times with PBS containing 5 mM EDTA and transferred to 96-well U-bottom plates. Dead cells were excluded by staining with ViaKrome 808 Fixable Viability Dye (Beckman Coulter, Woerden, The Netherlands) for 30 min on ice. Afterwards, cells were washed twice and fixed with 4% paraformaldehyde (Alfa Aesar, Kandel, Germany) for 15 min at room temperature. Finally, at least 10,000 events per sample were analyzed using a CytoFLEX LX flow cytometer (Beckman Coulter Inc., CA, USA) equipped with a 638 nm laser and a 660/10 fluorescent channel. Data were processed using FlowJo Software v.10.5 (FlowJo LCC, Ashland, USA) and GraphPad Prism 8.3.0 (Graphpad Software LLC., San Diego, USA). In the resulting histograms, the first fluorescent peak was identified as cells containing a single bead. Average bead uptake and fold change in bead uptake per cell was calculated based on established formulas from the literature^11,30^.

$${\text{Bead/cell = }}\frac{{{\mathrm{MFI}}_{{{\mathrm{total}}}} }}{{{\mathrm{MFI}}_{{{\mathrm{1bead/cell}}}} }};\,\,{\text{fold~change = }}\frac{{{\mathrm{beads/cell}}_{{{\mathrm{M1~or~M2}}}} }}{{{\mathrm{beads/cell}}_{{{\mathrm{undifferentiated~cell}}}} }}$$

### Assessment of bead internalization by Confocal microscopy

To assess bead internalization, macrophages were placed at a density of 2 × 10^5^ cells/well in a 24-well plate containing sterilized 12 mm coverslips. After differentiation for 24, 48, or 72 h, cells were co-cultured with FluoSpheres fluorescent beads at a bead-to-cell ratio of 10:1 for 4 h. Following incubation, macrophages were washed three times with cold Hank’s balanced salt solution (HBSS) (Gibco, Paisley, UK) to remove non-phagocytosed beads. Macrophage membranes were then stained with 2 μg/ml Alexa Fluor 488-conjugated Wheat germ agglutinin (WGA) (Life Technologies Corporation, Eugene, USA) in HBSS for 10 min at 37 °C. Subsequently, WGA was removed, and macrophages were washed twice with HBSS and fixed with 4% paraformaldehyde for 15 min at room temperature. Coverslips with macrophages were mounted onto polysine slides (Thermo Scientific, Braunschweig, Germany) using FluorSave Reagent (Millipore, San Diego, USA). Finally, bead uptake and internalization were assessed using a Leica TCS SPE-II and LAS-AF software (Leica Microsystems B.V., Amsterdam, The Netherlands). Z-stack imaging was performed to verify complete bead internalization. The images from confocal microscopy were analyzed using Fiji imageJ.

### Statistical analysis

Statistical analyses were performed using GraphPad Prism 8.3.0 (Graphpad Software LLC., San Diego, USA). Two-way ANOVA tests with multiple comparisons were used to determine statistical differences. For datasets with missing values, a mixed-effects model was applied. Results are expressed as mean ± SD, and all experiments were independently repeated at least three times. P-values were adjusted for multiple comparisons, with significance levels defined as *p < 0.05 (significant) and **p < 0.01 (highly significant).

## Supplementary Information

Supplementary Information 1.

Supplementary Video 1.

Supplementary Video 2.

Supplementary Video 3.

Supplementary Video 4.

Supplementary Video 5.

Supplementary Video 6.

Supplementary Video 7.

Supplementary Video 8.

Supplementary Video 9.

## Data Availability

Data are available in article supplementary material. Raw data files are available on request from the authors.
