# Supplementary material for: A canine macrophage cell line that can be polarized provides a model for canine macrophage differentiation
Source: Sci Rep. 2026 May 29;16:24613. doi: 10.1038/s41598-026-52770-7 (PMC13454523; doi:10.1038/s41598-026-52770-7)
Supplement: Supplementary file 1 [file 41598_2026_52770_MOESM1_ESM.pdf]

Figure S1

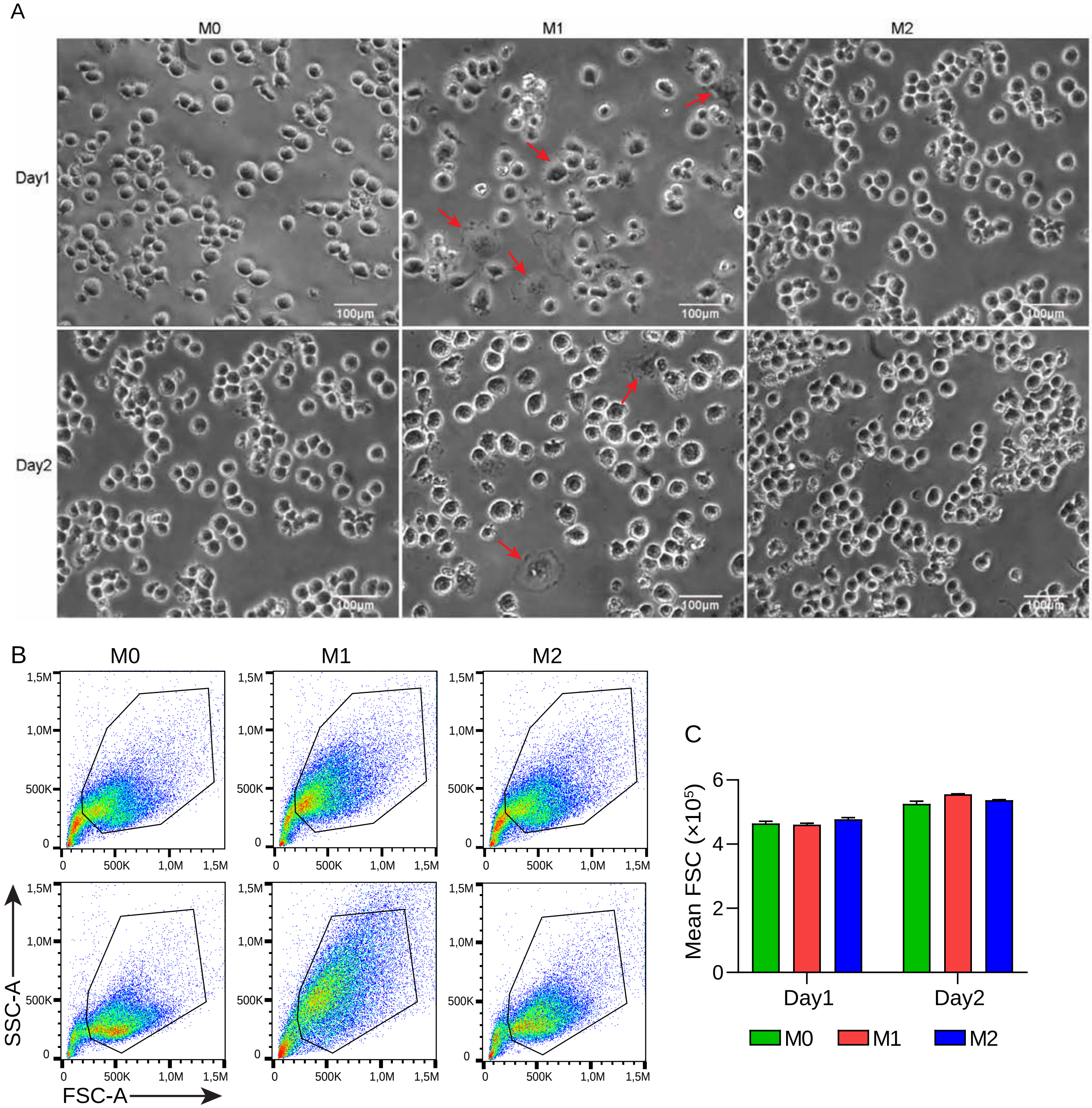

Supplementary Figure 1 Morphological changes in canine 030D-derived M0, M1 and M2 macrophages on day 1 and day 2. **(A)** Representative phase-contrast microscopy images (20× magnification) show morphological changes at day 1, and day 2. Large, rounded, or amoeboid morphology is indicated with red arrows. **(B)** Representative flow cytometry plots (FSC vs SSC) of macrophages at day 1 and day 2. **(C)** Mean FSC values quantify cell size differences among M0, M1, and M2 macrophages. Data are presented as mean ±SD, with n ⩾ 3 per condition. SSC: side scatter, FSC: forward scatter.

Supplementary Figure 2

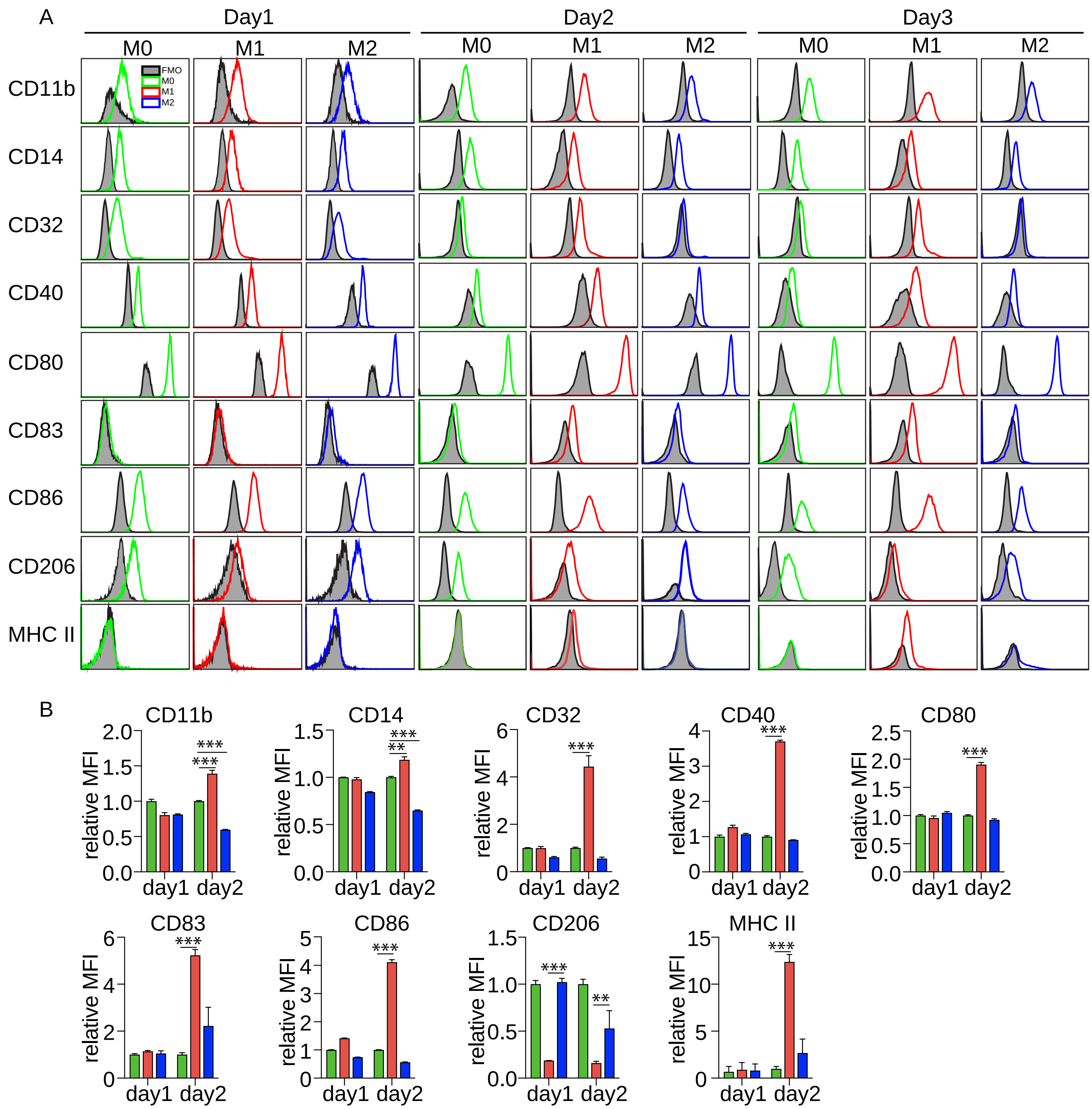

Supplementary Figure 2 Cell surface marker expression in 030D-derived macrophages over 3 days. **(A)** Representative histograms show expression of surface markers in M0, M1, and M2 macrophages at day1, 2 and 3. Fluorescence minus one (FMO) controls (gray histograms) were included to determine background fluorescence. **(B)** Expression levels were quantified at day 1 and day 2 (day 3 data are shown in the main figure). Relative MFI was calculated by determining the geometric mean fluorescence intensity for each sample, subtracting the corresponding FMO control, and normalizing the values to M0 cells. Data are presented as mean ±SD, with n⩾3 per condition. **p < 0.05; **p < 0.01, ***p < 0.001*.

Supplementary Figure 3

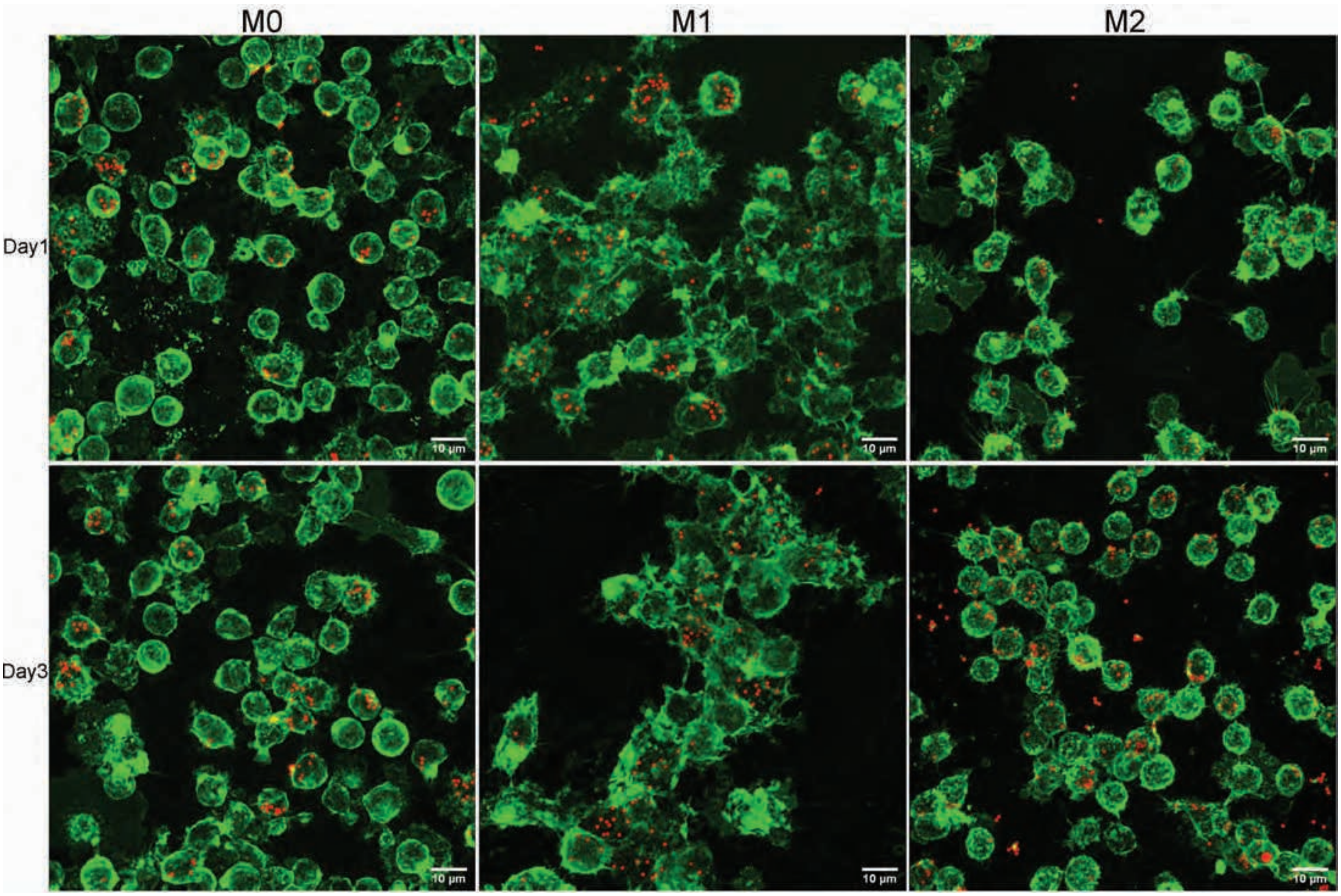

Supplementary Figure 3 Phagocytosis of canine 030D-derived M0, M1 and M2 macrophages at day 1 and 3. 030D cells were polarized for 1, or 3 days, followed by incubating with crimson carboxylate-modified FluoSpheres fluorescent beads. Phagocytosis by 030D-derived macrophages was confirmed by confocal microscopy. Plasma membranes were stained with wheat germ agglutinin (WGA, green) and beads are shown in red. Some extracellular beads are visible despite washing steps, likely representing non-internalized beads adhering to the cell surface or coverslip. Representative images of M0, M1, and M2 macrophages are shown at day 1 (top) and day 3 (bottom). 3D reconstructions based on z-stack illustrate the spatial relationship between macrophage and beads (see Supplementary materials Video S1A, B and C for day 1, and Video S3A, B and C for day 3). Scale bar=10 µm.
